# SLC7A11 inhibits ferroptosis and downregulates PD-L1 levels in lung adenocarcinoma

**DOI:** 10.3389/fimmu.2024.1372215

**Published:** 2024-04-09

**Authors:** Zhenyao Huang, Xia Chen, Yun Wang, Jiali Yuan, Jing Li, Wenlu Hang, Hao Meng

**Affiliations:** ^1^ Department of Respiratory and Critical Care Medicine, Second Affiliated Hospital of Xuzhou Medical University, Xuzhou, China; ^2^ Key Laboratory of Human Genetics and Environmental Medicine, School of Public Health, Xuzhou Medical University, Xuzhou, China; ^3^ Department of Respiratory Medicine, Xuyi People’s Hospital, Huai’an, Jiangsu, China; ^4^ Department of Dermatology, the Affiliated Huai'an Hospital of Xuzhou Medical University, the Second People's Hospital of Huai’an, Huai’an, China

**Keywords:** ferroptosis, LUAD, PD-L1, SLC7A11, immune cell infiltration

## Abstract

**Introduction:**

Lung adenocarcinoma (LUAD) is a prevalent form of lung cancer originating from lung glandular cells with low survival rates despite recent therapeutic advances due to its diverse and complex nature. Recent evidence suggests a link between ferroptosis and the effectiveness of anti-PD-L1 therapy, with potential synergistic effects.

**Methods:**

Our study comprehensively analyzed the expression patterns of ferroptosis regulators in LUAD and their association with prognosis and PD-L1 expression. Furthermore, we identified two distinct subtypes of LUAD through consensus clustering of ferroptosis regulators, revealing significant tumor heterogeneity, divergent PD-L1 expression, and varying prognoses between the subtypes.

**Results:**

Among the selected ferroptosis regulators, SLC7A11 emerged as an independent prognostic marker for LUAD patients and exhibited a negative correlation with PD-L1 expression. Subsequent investigations revealed high expression of SLC7A11 in the LUAD population. *In vitro* experiments demonstrated that overexpression of SLC7A11 led to reduced PD-L1 expression and inhibited ferroptosis in A549 cells, underscoring the significant role of SLC7A11 in LUAD. Additionally, pan-cancer analyses indicated an association between SLC7A11 and the expression of immune checkpoint genes across multiple cancer types with poor prognoses.

**Discussion:**

From a clinical standpoint, these findings offer a foundation for identifying and optimizing potential combination strategies to enhance the therapeutic effectiveness of immune checkpoint inhibitors and improve the prognosis of patients with LUAD.

## 1 Introduction

Lung adenocarcinoma (LUAD) is one of the most common forms of lung cancer that arises from the glandular cells of the lung (1, 2). It belongs to the group of non-small cell lung cancers (NSCLC), which are further separated into many subtypes and variants (3). Over the past few decades, LUAD has increasingly become the most common form of lung cancer, displacing squamous cell lung carcinoma in many countries (4). Despite recent therapeutic advances, the overall survival rate of LUAD patients remains low (5, 6). Furthermore, a number of molecularly targeted medications are unsuccessful in some LUAD patients since it is a very diverse tumor with complicated molecular pathways, which presents a significant obstacle to its therapy (7). The complexity and heterogeneity of LUAD present challenges in developing targeted therapies that can effectively combat the disease across different patient populations. Additionally, the low overall survival rate emphasizes the critical importance of advancing our understanding of LUAD’s molecular mechanisms and identifying new therapeutic targets. By doing so, we can hope to develop more personalized and effective treatment strategies that can improve outcomes for LUAD patients. This underscores the importance of ongoing research efforts aimed at uncovering novel targets and developing innovative medications to address the unique characteristics of LUAD and ultimately enhance patient prognosis.

Ferroptosis, a form of iron-dependent programmed cell death distinct from other controlled cell death mechanisms such as apoptosis, is marked by the accumulation of lipid peroxides (8). Tumor cells have been observed to possess the capability to suppress ferroptosis as a means to sustain their proliferative and invasive potential (9). The targeted induction of ferroptosis holds promise as a novel approach in cancer treatment, particularly in addressing the resistance of cancer cells to current chemotherapeutic agents (10). By elucidating the specific mechanisms through which ferroptosis is regulated and suppressed in tumor cells, researchers may uncover new avenues for developing targeted therapies that can effectively trigger ferroptosis and overcome cancer cell resistance. Furthermore, given the intricate molecular landscape of LUAD, investigating the role of ferroptosis-related genes in the prognosis of this cancer subtype is crucial for identifying potential prognostic markers and therapeutic targets. A deeper understanding of the interplay between ferroptosis and LUAD progression could pave the way for the development of more tailored and effective treatment strategies, ultimately improving outcomes for patients with this challenging disease.

Blocking the PD-1/PD-L1 axis through immune therapy has demonstrated long-lasting antitumor activity in multiple cancer types (11). The clinical triumph of anti–PD-1/PD-L1 therapy is largely ascribed to its ability to reinvigorate tumor antigen–specific T cells that have been rendered inactive by the interaction of PD-1 on T cells with its ligand PD-L1 on tumor cells (12). Nevertheless, the response rates remain suboptimal, with only approximately 10%–30% of patients deriving benefits from the therapy as a standalone treatment, primarily due to therapy resistance and the lack of adequate biomarkers for patient stratification (13). Recent evidence suggests that tumor-killing T cells and anti-PD-L1 antibodies induce ferroptosis in tumor cells, while ferroptosis inhibitors diminish the anticancer effectiveness of these agents (14). Furthermore, a synergistic effect has been observed between anti-PD-L1 antibodies and ferroptosis activators, effectively suppressing tumor growth in both preclinical and animal studies (15). Understanding the intricate interplay between ferroptosis and the tumor immune microenvironment in the context of LUAD is essential for unraveling the full therapeutic potential of this interaction. Elucidating the specific mechanisms through which ferroptosis is modulated in the tumor immune microenvironment and its impact on the response to immunotherapy could provide valuable insights for developing more effective treatment strategies (16). Therefore, exploring the potential of ferroptosis as a predictive biomarker for patient stratification in the context of anti–PD-1/PD-L1 therapy could offer new avenues for improving patient outcomes and refining treatment approaches in LUAD.

Here, we undertook a comprehensive analysis, delving into the expression profiles and their associations with prognosis, PD-L1, and roles within the tumor immune microenvironment of ferroptosis regulators in LUAD. Furthermore, we stratified subtypes based on the expression levels of these regulators, revealing significant tumor heterogeneity, distinct PD-L1 expression, and variations in the tumor immune microenvironment between the identified subtypes. These findings hold promise for aiding in risk stratification and facilitating precision therapy for patients with LUAD. Subsequently, our investigation pinpointed SLC7A11 as a potential immune infiltration-related ferroptosis regulator, with high expression correlating with poor prognosis and exhibiting a negative correlation with PD-L1 expression in LUAD. Moreover, we scrutinized the expression profiles of SLC7A11 in clinical tissue samples and probed into its potential roles and functions in regulating ferroptosis in LUAD cell lines. Additionally, our study comprehensively explored the pan-cancer expression profiles, prognostic significance, and associations with immune checkpoints of SLC7A11. These findings provide novel insights into the regulatory mechanisms intertwined with the tumor immune microenvironment and offer potential avenues for advancing immunotherapeutic strategies for LUAD.

## 2 Method

### 2.1 Data collection

The RNA-seq data and clinical information for the LUAD patients were obtained from the Genomic Data Commons (GDC) data portal of the Cancer Genome Atlas (TCGA) database (https://portal.gdc.com). This dataset included 516 LUAD tissues and 59 paracancerous tissues, and the clinical characteristics of the patients in this study can be found in **Supplementary Table 1**. The genes related to ferroptosis are sourced from Ze-Xian Liu et al.’s systematic analysis of the aberrations and functions of ferroptosis in cancer (17). To further validate the expression level of target genes, the GSE46539 datasets from the Gene Expression Omnibus database (GEO) were downloaded and utilized (https://www.ncbi.nlm.nih.gov/geo/).

### 2.2 Bioinformatics analysis

R version 4.0.3 was used to carry out each of the previously listed analytical techniques. A consistency study was performed using a maximum of 6 clusters, 100 repeats, 80% of the total samples randomly picked each time, clusterAlg = “hc”, and innerLinkage = “ward.D2” using the R package ConsensusClusterPlus (v1.54.0). The R program pheatmap (v1.0.12) was used to evaluate the clustering heatmaps, and genes with a variance over 0.1 were retained in the gene expression heatmaps. The top 25% of genes sorted by variance from greatest to lowest were presented if the input target gene count was more than 1000. PCA plots were produced with the R program ggord, and box plots were produced with ggplot2. In the Kaplan-Meier survival study, the differences in survival between the two groups stated above were examined using the log-rank test. To examine the target genes’ prediction accuracy, TimeROC analysis was performed. Log-rank tests and univariate Cox regression were used to get the p-values and hazard ratios (HR) with 95% confidence intervals (CI) for the Kaplan-Meier curve. To examine the effects of smoking, alcohol intake, target genes, and subtypes on LUAD risk, univariate Cox regression analysis was employed. The R package “forestplot” was used to create a forest plot that displayed the p-values, HR, and 95% CI.

### 2.3 Human clinical samples

A total of 37 pairs of LUAD and adjacent normal tissues and blood were collected from patients who underwent radical nephrectomy at the Second Affiliated Hospital of Xuzhou Medical University between March and December 2015. Written informed consent was obtained from each patient, and the Institutional Ethics Committee at the hospital approved the study. Total RNA was extracted using TRIzol reagent (Takara) and reverse-transcribed into cDNA using the PrimeScript RT Reagent Kit (Takara). The relative expression of genes was determined by RT-qPCR using the SYBR Premix Ex Taq (Takara) and the ABI 7500 fast real-time PCR System (Applied Biosystems), with GAPDH serving as an endogenous normalization reference. The clinical information of all patients and the primers used are provided in **Supplementary Tables 2**, **3**.

### 2.4 Cell experiments

The A549 human alveolar basal epithelial cancer cell line and BEAS-2B normal human lung bronchial epithelial cells were obtained from the Shanghai Institute of Cell Biology. To overexpress SLC7A11 via plasmid transfection, the plasmid containing the target gene is introduced into the cells using transfection reagent. To knockdown SLC7A11 in A549, a specific small interfering RNA (siRNA) targeting the gene is transfected into the cells using transfection reagents. Cell function assays, such as cell viability, cell migration, and ROS detection, were conducted using established research methods (18, 19). The steps for cell viability CCK-8 assay involve seeding cells, treating them, adding CCK-8 reagent, incubating, reading absorbance, and analyzing data to assess cell viability and proliferation effects. To assess cell migration using the Transwell assay, cells are plated in the upper chamber of a Transwell insert, while a chemoattractant or conditioned media is placed in the lower chamber. After incubation, cells that migrate through the porous membrane to the lower chamber are quantified, providing insights into their migratory capacity. The iron concentration was measured using an iron assay kit (Abcam, ab83366) following the manufacturer’s instructions. The detection of cellular ROS using fluorescence microscopy involves labeling cells with ROS-sensitive fluorescent probes, followed by visualizing the fluorescence signal emitted by the probes to assess the levels of ROS within the cells. The Western blot experiment included sample protein extraction, separation of proteins by gel electrophoresis, antibody detection of PD-L1 (Abcam, ab205921), and analysis of protein expression levels through band intensity quantification.

### 2.5 Pan-cancer analysis

For the pan-cancer investigation of SLC7A11, RNA-seq data and accompanying clinical information for 33 cancer types were also gathered from TCGA. The abbreviations for numerous cancer types and ferroptosis regulators in this study can be found in **Supplementary Tables 4**, **5**. To undertake a reliable evaluation of immune-related correlations, we deployed immunedeconv, a R software package that includes six state-of-the-art algorithms, including TIMER, xCell, MCP-counter, CIBERSORT, EPIC, and quanTIseq. To examine the expression of these eight immune checkpoint-related genes, transcripts corresponding to SIGLEC15, IDO1, PD-L1, HAVCR2, PDCD1, CTLA4, LAG3, and PDCD1LG2 were extracted. R software version 4.0.3 was used to conduct the statistical analysis. *p* < 0.05 was deemed statistically significant when comparing the two sets of data using the Wilcoxon rank-sum test.

### 2.6 Statistical analysis

We used GraphPad Prism 8.0 and R, version 4.0.3, to conduct statistical tests. The Student’s *t*-test was used to compare two groups of continuous variables. Differences in proportions were compared by the Chi-squared test. For comparing more than two groups, the one-way ANOVA or Kruskal-Wallis test were utilized. Kaplan-Meier analysis were utilized with the log-rank test to compare the overall survival (OS) and progression-free survival between different groups. Additionally, univariate and multivariate Cox regression analyses were to conducted identify independent factors associated with OS. A statistical significance threshold of *p* < 0.05 was applied to ascertain the results. Mechanism figure is drawn by Figdraw.

## 3 Results

### 3.1 Ferroptosis regulator gene expression differences between adjacent normal tissues and LUAD

**Figure 1:**
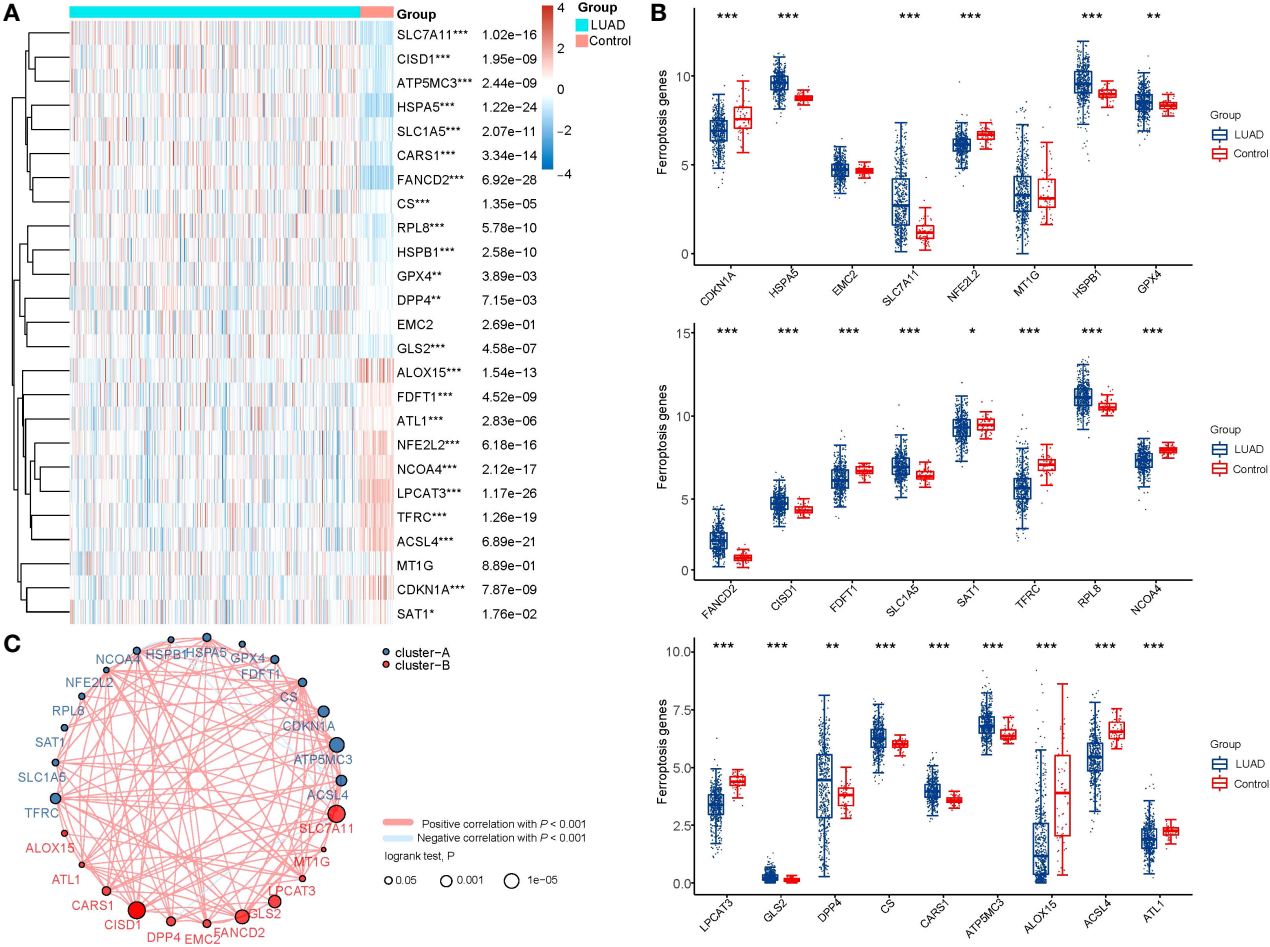
The expression distribution, correlation, and prognostic significance of 25 ferroptosis regulator genes in LUAD patients in TCGA database. **(A)** Heatmap analysis and **(B)** Scatter plot of the expression of 25 ferroptosis regulator genes between LUAD patients and normal controls. **(C)** Spearman correlation and prognostic significance of ferroptosis regulators in LUAD. The red and blue lines represent positive and negative correlations, respectively. The red and blue dots represent poor and good prognosis, respectively. The larger the circle, the lower the prognosis log rank. *p < 0.05, **p < 0.01, and ***p < 0.001.

A total of 44 ferroptosis regulator genes identified from prior studies were deemed essential ferroptosis regulators because of their critical functions in controlling ferroptosis (8, 17). Using the TCGA dataset, the expression patterns of a few chosen ferroptosis regulators between LUAD and nearby normal pairs were methodically investigated in order to assess the biological roles of these regulators in LUAD. The expression profiles of 59 normal patients and 516 instances of LUAD individuals were retrieved and examined. We screened a total of 1103 differentially expressed genes in LUAD, and 25 out of 44 ferroptosis regulator genes were among them, so we included these 25 genes as key ferroptosis regulator genes in LUAD in the subsequent study (**Supplementary Figure 1**). In further screening, our analysis revealed distinct expression levels of 25 ferroptosis regulators in LUAD and normal tissues (**Figures 1A, B**). Specifically, we found upregulated ferroptosis regulators HSPA5, SLC7A11, HSPB1, GPX4, FANCD2, CISD1, SLC1A5, RPL8, GLS2, DPP4, CS, CARS1, and ATP5MC3 (*p*<0.05), as well as downregulated regulators CDKN1A, NFE2L2, FDFT1, SAT1, TFRC, NCOA4, LPCAT3, ALOX15, ACSL4, and ATL1 (*p*<0.05). However, no significant differences were observed between LUAD and normal tissues in the expression of EMC2 and MT1G (*p*>0.05). Furthermore, correlation and prognosis analysis demonstrated that the expression of most ferroptosis regulatory genes was positively correlated and played a crucial prognostic role in LUAD (**Figure 1C**). These findings suggest that ferroptosis regulator genes may play a critical role in regulating tumorigenesis and the development of LUAD.

### 3.2 Ferroptosis regulator consensus clustering analysis revealed notable variations in baseline characteristics and survival across two patient clusters

**Figure 2:**
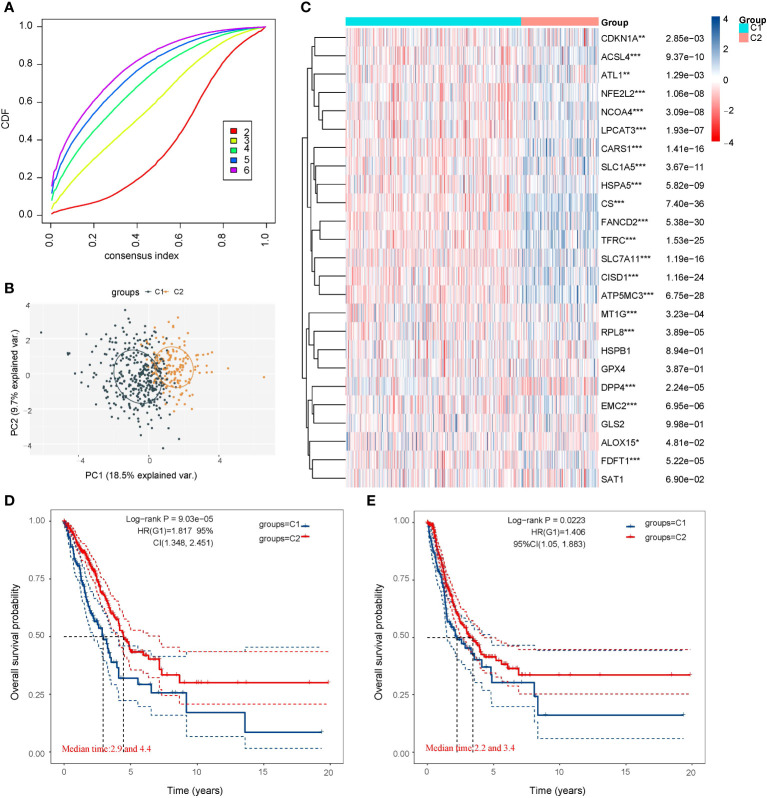
LUAD cases in the TCGA database were artificially divided into two subtypes for comparison. **(A)** Curves of the cumulative distribution function for k = 2–6. **(B)** Principal component analysis of the two subtypes showed that they could be better distinguished. **(C)** Heatmap of 25 ferroptosis regulator genes expression of the two subtypes. Most of the ferroptosis regulators were highly or lowly expressed in cluster 1, while there was no difference in the expression of four regulators (HSPB1, GPX4, GLS2, and SAT1) between the two clusters. **(D)** Comparison of the Overall Survival Kaplan–Meier curves for the two subtypes. **(E)** Comparison of the Disease Free Survival Kaplan–Meier curves for the two subtypes. Cluster 1 was significantly associated with a lower tumor stage and cancer grade. *p < 0.05, **p < 0.01, and ***p < 0.001.

We utilized the Consensus Cluster Plus package of R software for consistency analysis. Based on the expression levels of selected ferroptosis regulators and the proportion of ambiguous clustering measures, we identified k = 2 as the optimal clustering stability from k = 2 to 6 (**Figure 2A**; **Supplementary Figure 2**). Consequently, the 516 LUAD patients were categorized into two subtypes, namely, cluster 1 (n = 358) and cluster 2 (n = 158). To validate the clustering results based on the expression of ferroptosis regulators, we then employed the PCA method to analyze the gene expression profiles between the two subtypes (**Figure 2B**). The gene expression profiles between the two subtypes exhibited distinct differences. Most of the ferroptosis regulators were highly or lowly expressed in cluster 1, while there was no difference in the expression of four regulators (HSPB1, GPX4, GLS2, and SAT1) between the two clusters (**Figure 2C**). Subsequently, differences were observed in the clinicopathological characteristics and prognosis between the two subtypes, as shown in **Supplementary Table 1**. Cluster 1 was significantly associated with a lower tumor stage and cancer grade (*p* < 0.01), while no statistical differences in age, sex, or race were found between the two clusters (*p*>0.05). Additionally, cluster 1 patients exhibited better overall survival (*p*< 0.001) and progression-free survival (*p*<0.001) than cluster 2 (**Figures 2D, E**). These results indicate significant heterogeneity between the two subtypes of LUAD patients.

### 3.3 Correlation between ferroptosis regulators, PD-L1 expression, and immune cell infiltration in LUAD

**Figure 3:**
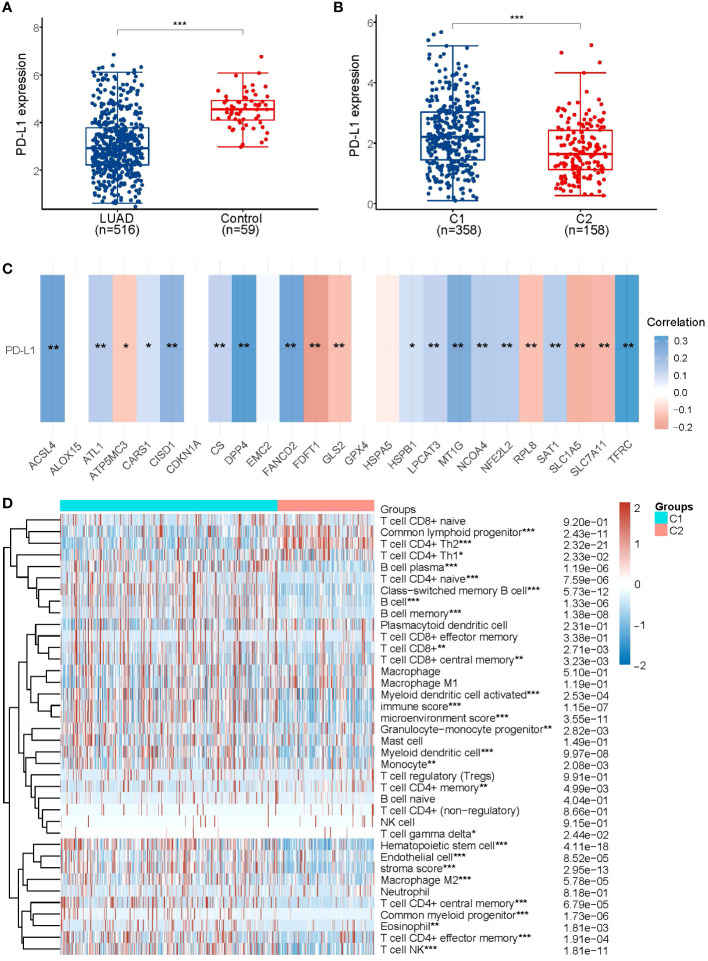
Association of PD-L1 with ferroptosis regulator genes. **(A)** PD-L1 is lowly expressed in LUAD patients compared to normal controls. **(B)** PD-L1 is lowly expressed in the poorer prognostic LUAD subtype, compared to the better prognostic subtype. **(C)** Heatmap of the correlation between PD-L1 and 25 ferroptosis regulator genes, blue represents positive correlation and red represents negative correlation. **(D)** Levels of infiltration of various immune cell types in the two subtypes of LUAD in TCGA. *p < 0.05, **p < 0.01, and ***p < 0.001.

In order to explore the relationship between PD-L1 and ferroptosis in LUAD, this study examined the divergent expression in two clusters and the association of PD-L1 with the ferroptosis regulators in LUAD. Unlike normal adjacent tissues and cluster 1 patients, the expression levels of PD-L1 in LUAD tissues and cluster 2 patients were notably low (*p*< 0.01; **Figures 3A, B**). An analysis involving 516 LUAD individuals revealed that PD-L1 exhibited an association with the expression levels of most ferroptosis regulators (**Figure 3C**). We further investigated the impact of ferroptosis regulators on the tumor immune microenvironment of LUAD. The two subtypes classified based on the expression levels of selected ferroptosis regulators showed significant differences in immune cell infiltration (**Figure 3D**). Cluster 2 exhibited higher levels of infiltrated activated Common lymphoid progenitor, T cell CD4+ Th2, T cell CD4+ Th1, while cluster 1 was more correlated with the B cell plasma, T cell CD4+ naive, B cell, B cell memory (**Figure 3D**; **Supplementary Figure 3**). The GSEA method was utilized to elucidate the underlying regulatory mechanisms causing the difference between the two clusters. Gene set enrichment analysis indicated that pentose phosphate, ERBB signaling, and alpha linolenic acid metabolism pathways are significantly enriched in cluster 1 (**Supplementary Figure 4A**).

### 3.4 The crucial ferroptosis regulator SLC7A11 exhibits upregulation in LUAD

**Figure 4:**
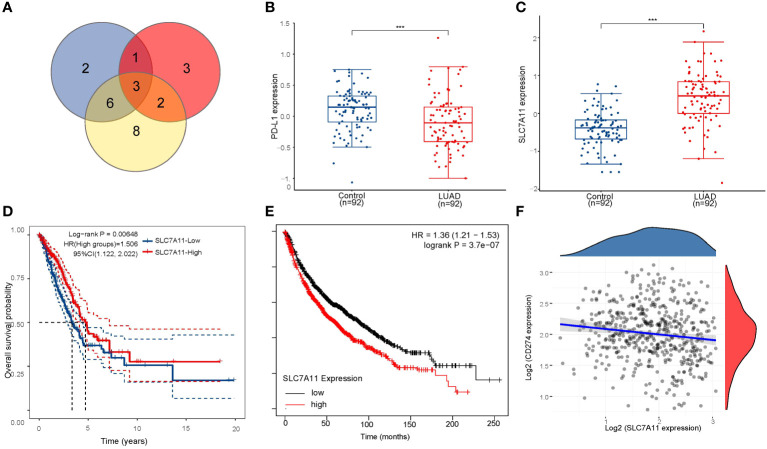
Screening for critical ferroptosis regulator genes. **(A)** The venn diagram screens for 3 genes associated with PD-L1 (DPP4, GLS2 and SLC7A11). **(B)** PD-L1 expression was significantly lower in LUAD patients than in controls in GSE46539 datasets. **(C)** SLC7A11 is highly expressed in LUAD patients in GSE46539 datasets. **(D)** The Kaplan–Meier analysis of LUAD patients with high and low SLC7A11 expression level in the TCGA database. **(E)** The Kaplan–Meier analysis of LUAD patients with high and low SLC7A11 expression level in Kaplan–Meier plotter database. **(F)** Spearman correlation analysis of SLC7A11 expression and PD-L1 expression in LUAD. ***p < 0.001.

As previously mentioned, the two subtypes of LUAD, distinguished by the levels of ferroptosis regulators, exhibited distinct PD-L1 expression and tumor immune environments. These findings indicate the crucial role of ferroptosis regulators in tumor development and immune infiltration. By identifying the highly expressed ferroptosis regulators in LUAD associated with poor prognosis and negatively correlated with PD-L1 expression, we identified SLC7A11, DPP4, and GLS2 as potential key poor prognostic ferroptosis regulators differently expressed in LUAD and correlated with PD-L1 expression (**Figure 4A**). To further illustrate the expression of SLC7A11, DPP4, and GLS2 in LUAD, we analyzed expression data from the GEO database. The results from the GSE46539 datasets showed significantly higher expression of SLC7A11, DPP4, and GLS2 in LUAD compared to normal tissues, while PD-L1 was expressed at lower levels (*p* < 0.05, **Figures 4B, C**; **Supplementary Figures 5A, D**). Additionally, the prognostic value of highly expressed SLC7A11, DPP4, and GLS2 was analyzed in LUAD, revealing that only the upregulated SLC7A11 group exhibited worse overall survival compared to the lowly expressed group (*p*<0.05, **Figure 4D**; **Supplementary Figures 5B, E**). This result was further confirmed in an independent LUAD cohort from the Kaplan–Meier plotter (*p*<0.05, **Figure 4E**; **Supplementary Figures 5C, F**). Similarly, LUAD tissue had higher levels of SLC7A11 protein expression than normal tissue did in the human protein atlas (**Supplementary Figure 6**). Further correlation analysis demonstrated a significant negative correlation between SLC7A11 and PD-L1 expression in LUAD (*p*<0.001, Spearman =-0.15) (**Figure 4F**). Therefore, SLC7A11 emerged as the sole highly expressed ferroptosis regulator in LUAD and cluster 2, exhibiting a negative correlation with PD-L1 expression and worse overall survival.

**Figure 5:**
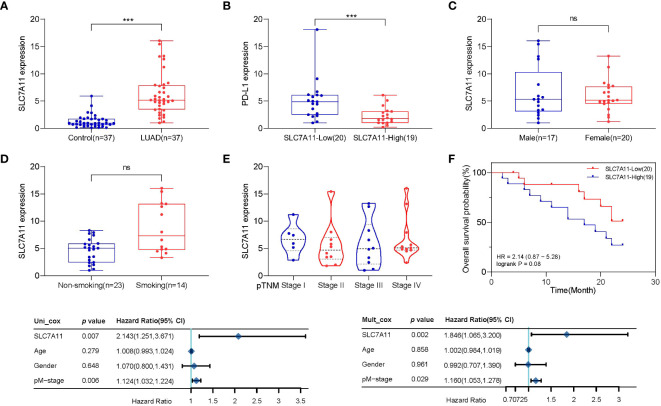
Population validation results for SLC7A11. **(A)** SLC7A11 levels were significantly higher in LUAD patients than in controls. **(B)** PD-L1 levels were significantly lower in LUAD patients than in controls. **(C)** There was no difference in SLC7A11 expression between LUAD patients of different sexes. **(D)** SLC7A11 expression was significantly higher in smoking LUAD patients than in non-smoking patients. **(E)** There was no statistically significant difference in SLC7A11 levels of LUAD patients with different stages. **(F)** The Kaplan–Meier survival analysis of LUAD patients with high and low SLC7A11 expression level in the validation population. **(G)** Forest plots based upon the outcomes of univariate Cox regression of SLC7A11 expression. **(H)** Forest plots based upon the outcomes of multivariate Cox regression. ***p < 0.001 and ns *p* > 0.05.

Subsequently, we obtained tumor samples from 37 patients with LUAD who underwent monthly follow-up visits. We assessed the expression of SLC7A11 and PD-L1 in the lung tissues of LUAD patients and normal paraneoplastic tissues (control) (**Figures 5A, B**). Our findings indicated high expression of SLC7A11 in LUAD and low expression of PD-L1, consistent with the previous section. Furthermore, there were no statistically significant differences in SLC7A11 expression across smoking, gender, or clinical stage (**Figures 5C-E**). Subsequently, we categorized the 37 LUAD patients into high and low SLC7A11 expression groups based on SCL7A11 expression in tumor tissue and plotted Kaplan–Meier curves (**Figure 5F**). Similarly, the group with upregulated SLC7A11 exhibited a trend toward worse overall survival compared to the lowly expressed group (logrank *p*=0.08). We also employed the Cox analysis method to investigate the relationship between SLC7A11 expression and overall survival in LUAD. Univariate analysis revealed a significant correlation between pM-stage (*p*<0.01) and SLC7A11 expression (*p*<0.01) with overall survival (**Figure 5G**). Furthermore, multivariate analysis demonstrated that SLC7A11 expression (*p*< 0.01) is an independent prognostic factor in LUAD patients (**Figure 5H**).

### 3.5 Effects of high expression of SLC7A11 in LUAD Cell Lines *in vitro*

**Figure 6:**
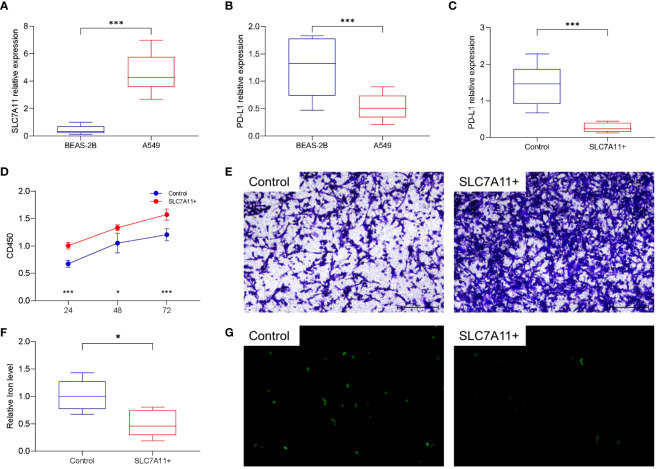
Cellular functional validation of SLC7A11. **(A)** SLC7A11 was highly expressed in lung cancer cell lines (A549) compared to normal lung tissue cell lines (BEAS-2B). **(B)** PD-L1 is lowly expressed in A549 cells compared to BEAS-2B cells. **(C)** Overexpression of SLC7A11 decreased PD-L1 expression in A549 cells. **(D)** Overexpression of SLC7A11 resulted in accelerated proliferation of A549 cells. **(E)** Overexpression of SLC7A11 enhanced the migration of A549 cells. **(F)** Overexpression of SLC7A11 reduced the level of cellular iron level. **(G)** Overexpression of SLC7A11 reduced the level of ROS. *p < 0.05, and ***p < 0.001.

Human LUAD cell lines A549 and normal lung epithelial cells BEAS-2B were used to detect the expression levels of SLC7A11 and PD-L1. Compared with normal cells, lung cancer cells A549 had higher levels of SLC7A11 expression and lower levels of PD-L1 expression, and the difference was statistically significant (**Figures 6A, B**). Meanwhile, we overexpressed SLC7A11 in A549, which resulted in reduced PD-L1 expression (**Figure 6C**). In contrast, knockdown of SLC7A11 showed a increase in PD-L1 expression in A549 (**Supplementary Figure 7A**). The expression level of PD-L1 protein after SLC overexpression or knockdown was explored using Western Blot, and the results were consistent with qRT-PCR (**Supplementary Figure 7B**). Overexpression of SLC7A11 also leads to an accelerated proliferation rate and migration of A549 cells (**Figures 6D, E**). We next explored the effect of overexpression of SLC7A11 on ferroptosis levels, overexpression of SLC7A11 resulted in lower iron ion concentration and reduced ROS accumulation in A549 cells (**Figures 6F, G**). All of these results suggest that high expression of SLC7A11 inhibits the ferroptosis levels of lung cancer cells, leading to accelerated proliferation and migration, which may be associated with poor prognosis.

### 3.6 Comprehensive analysis of key ferroptosis regulator SLC7A11 in pan-cancer

**Figure 7:**
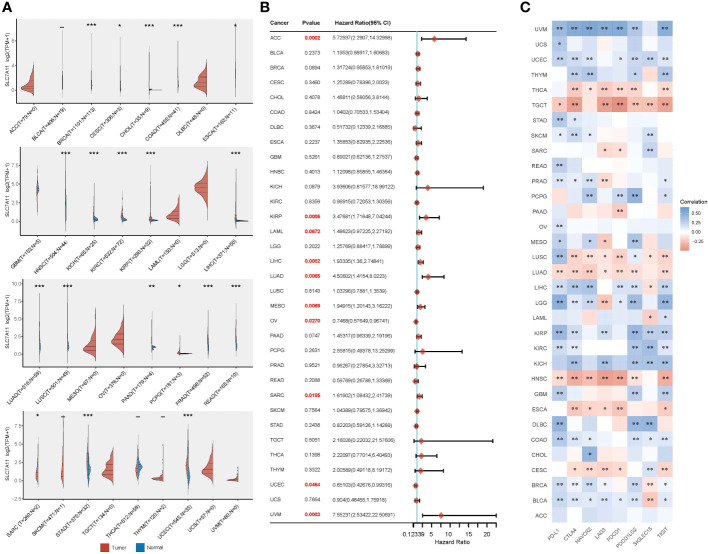
Comprehensive analysis of the crucial ferroptosis regulator SLC7A11 across different types of cancer. **(A)** SLC7A11 exhibited varying levels of expression in the majority of cancer types. **(B)** Elevated SLC7A11 expression was linked to lower overall survival rates in different cancer types. **(C)** The correlation between SLC7A11 expression and the expression of eight common immune checkpoints was evident in most cancer types, particularly in relation to PD-L1 expression. *p < 0.05, **p < 0.01, and ***p < 0.001.

Given the widespread involvement of ferroptosis and immune infiltration in the development of various cancer types, we aimed to investigate whether SLC7A11, the key ferroptosis regulator in our study, also predicts poor prognosis and influences the tumor immune microenvironment in other cancer types. To begin, we analyzed the relative expression of SLC7A11 in over 10,000 tumor and normal tissue samples in the TCGA and GTEx databases. The results revealed that, compared to normal tissues, SLC7A11 was significantly upregulated in 11 cancer types (BRCA, CESC, ESCA, HNSC, LUAD, LUSC, PRAD, READ, SARC, STAD, and UCEC) and downregulated in 9 cancer types (CHOL, COAD, KICH, KIRC, KIRP, LIHC, PAAD, PCPG, and THCA) (*p*< 0.05; **Figure 7A**). Furthermore, we investigated the prognostic significance of SLC7A11 across multiple cancer types. Univariate Cox regression analyses in pan-cancer indicated that upregulation of SLC7A11 was associated with poor overall survival in 10 cancer types (ACC, KIRP, LAML, LIHC, LUAD, MESO, OV, SARC, UCEC, UVM) (*p*< 0.05; **Figure 7B**). Additionally, we conducted an expression correlation analysis between SLC7A11 and the eight most common immune checkpoint-related genes (including PD-L1, CTLA4, HAVCR2, LAG3, PDCD1, PDCD1LG2, SIGLEC15, and TIGIT). The results indicated a close relationship between SLC7A11 and the expression of checkpoint-related genes in most cancer types, particularly PD-L1 expression (**Figure 7C**). In summary, high expression of SLC7A11 was associated with the expression of immune checkpoint-related genes and poor prognosis in various prevalent cancers.

## 4 Discussion

Ferroptosis, characterized by membrane rupture and cell death resulting from the accumulation of iron-dependent lipid peroxides, has emerged as a significant process in cancer biology (20). The use of ferroptosis inducers or the modulation of ferroptosis-regulated genes has shown promise in inhibiting tumor cell growth and overcoming drug resistance, particularly in cancers such as LUAD, where evasion of apoptosis is a challenge (21). Notably, ferroptosis has been linked to KRAS mutations, epithelial mesenchymal transition, and epidermal growth factor receptor mutations, underscoring its involvement in the development and progression of LUAD (5, 22–24). Moreover, the identification of ferroptosis inhibitors associated with poor prognosis and tumor cell proliferation in LUAD further emphasizes the complex role of ferroptosis in both the pathogenesis of LUAD and its potential as a target for anticancer therapy (25, 26). In our study, we employed consensus clustering to delineate two distinct subtypes characterized by differential clinical features, prognoses, and PD-L1 expression based on selected ferroptosis regulators. Notably, among these regulators, SLC7A11 emerged as a potential immune infiltration-related ferroptosis regulator, shedding light on its role in the tumor microenvironment. Furthermore, our pan-cancer analysis unveiled a close association between SLC7A11 expression, PD-L1 expression, and poor overall survival across multiple cancer types, indicating the broader implications of SLC7A11 and ferroptosis in cancer beyond LUAD. This underscores the need for further investigation into the intricate interplay between ferroptosis, immune regulation, and cancer progression, with potential implications for the development of novel therapeutic strategies.

The programmed death-1 (PD-1) pathway is a pivotal regulator of local immunosuppression within the tumor microenvironment, orchestrating the activation of T-cells against tumor antigens in secondary lymph nodes (27, 28). Disrupting this pathway by blocking PD-1 receptors on immune cells or PD-L1 ligands on tumor and/or immune cells has emerged as a promising strategy for inhibiting tumor growth and potentially achieving a curative effect. However, our research uncovered a predominant low expression of PD-L1 in patients with LUAD, associated with high expression of SLC7A11, leading to a significant clinical challenge. Specifically, individuals with “Low PD-L1 expression” lung cancer often lack effective first-line single drug therapy, as their response to immune checkpoint inhibitors is notably limited (29). While combining anti PD-1 antibody therapy with conventional treatments has shown potential for improving treatment response, the scarcity of available drugs remains a substantial hurdle in clinical practice (30). This scarcity underscores the ongoing challenge of striking a delicate balance between treatment toxicity and therapeutic benefit. As such, the need for novel therapeutic approaches that can effectively address the limitations associated with low PD-L1 expression in LUAD patients is increasingly apparent.

Through cellular experiments, we demonstrated that high expression of SLC7A11 in LUAD cells suppresses ferroptosis (lower iron ion concentration and reduced ROS accumulation). At the meantime, overexpression of SLC7A11 leads to decreased expression of PD-L1. These results all suggest a strong link between SLC7A11-mediated ferroptosis and PD-L1. The correlation between ferroptosis and the PD-1/PD-L1 immune checkpoint has become a focal point in biomedical research (31, 32). Despite stemming from distinct biological pathways, these phenomena hold potential implications for tumor treatment and immune modulation. Recent studies suggest that ferroptosis may play a role in tumor immune evasion (33, 34). Disruptions in iron metabolism could impact how tumor cells are recognized by the immune system and their susceptibility to immune attacks, thereby influencing the regulatory functions of the PD-1/PD-L1 immune checkpoint (35, 36). Consequently, gaining insights into the interplay between ferroptosis and PD-1/PD-L1 immune regulation holds promise for advancing the development of more efficacious strategies for treating tumors.

Research has demonstrated that immunotherapy-activated CD8+ T cells can heighten iron death-specific lipid peroxidation in tumor cells, thereby augmenting the anti-tumor efficacy of immunotherapy (37). The release of IFNγ by CD8+ T cells has been found to down-regulate the expression of SLC7A11, thereby restricting cystine uptake by tumor cells and promoting tumor cell lipid peroxidation and ferroptosis. The SLC7A11-GSH-GPX4 pathway, recognized as the most crucial and earliest discovered antioxidant system, plays a pivotal role in this process (38). Notably, the overexpression of SLC7A11 leads to heightened consumption of glutamine for glutathione synthesis, a hallmark of glutamine addiction in cancer cells (39). This metabolic reprogramming, characterized by elevated glucose consumption, lactate production, and glutamine addiction, highlights the pivotal role of SLC7A11 as a critical ferroptosis regulator in the context of cancer metabolism (40). By targeting SLC7A11 and its associated pathways, novel therapeutic strategies can be developed to disrupt the metabolic vulnerabilities of cancer cells, potentially leading to more effective treatments for LUAD and other cancers. This underscores the potential of SLC7A11 as a druggable target and emphasizes the importance of further exploration into the development of precision therapies aimed at disrupting the metabolic dependencies of cancer cells.

**Figure 8:**
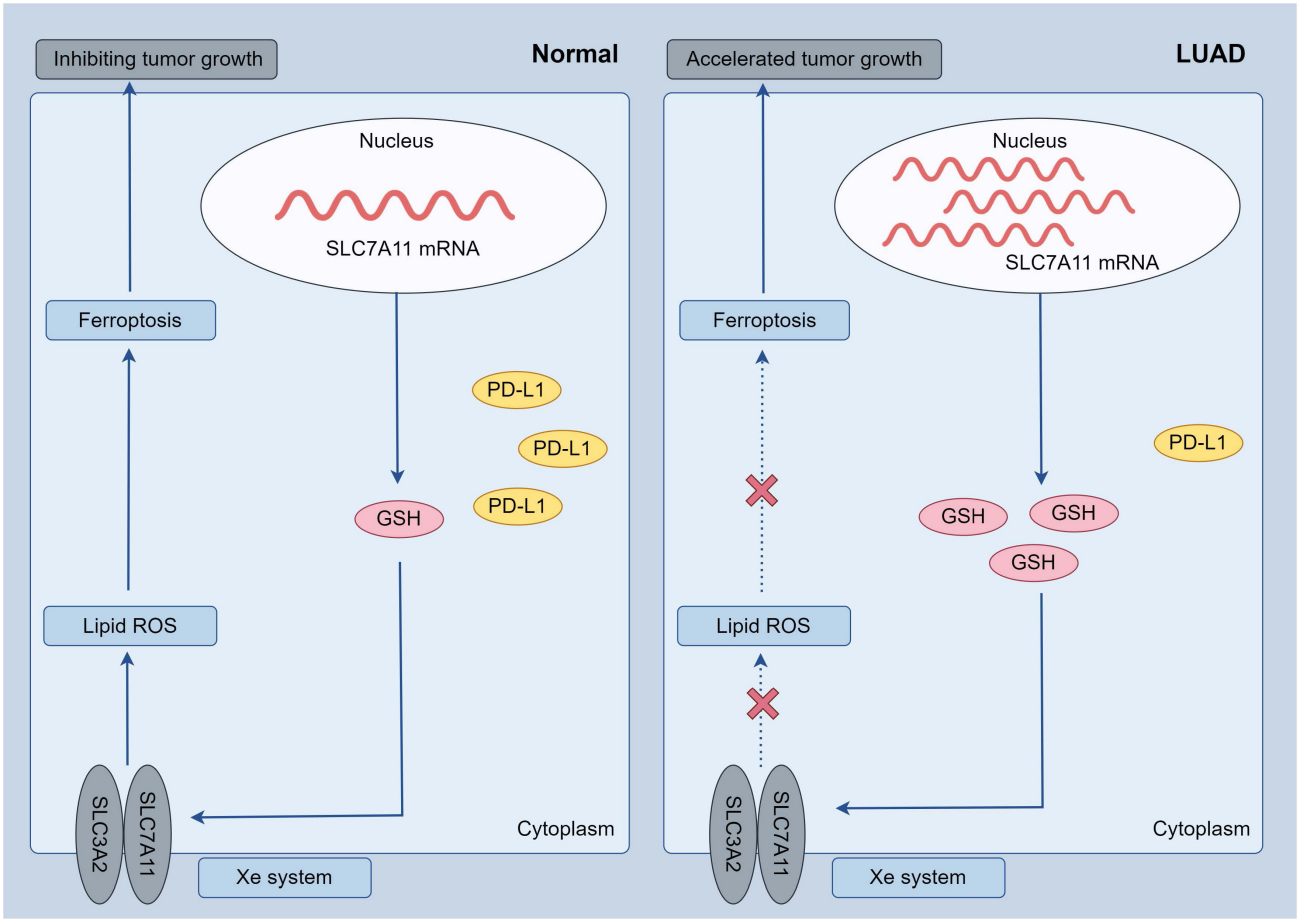
Possible mechanisms: in LUAD tumor cells, increased SLC7A11 expression resulted in decreased PD-L1 and suppressed ferroptosis (increased GSH, decreased oxidative stress), further leading to accelerated tumor cell growth.

In summary, this study comprehensively analyzed the expression patterns of ferroptosis regulators in LUAD and their association with prognosis and PD-L1 expression. Furthermore, we identified two distinct subtypes of LUAD through consensus clustering of ferroptosis regulators, revealing significant tumor heterogeneity, divergent PD-L1 expression, and varying prognoses between the subtypes. These findings have the potential to aid in risk stratification and the precise treatment of LUAD patients. Among the selected ferroptosis regulators, SLC7A11 emerged as an independent prognostic marker for LUAD patients and exhibited a negative correlation with PD-L1 expression. Subsequent investigations revealed high expression of SLC7A11 in the LUAD population. *In vitro* experiments demonstrated that overexpression of SLC7A11 led to reduced PD-L1 expression and inhibited ferroptosis in A549 cells, underscoring the significant role of SLC7A11 in LUAD (**Figure 8**). Additionally, pan-cancer analyses indicated an association between SLC7A11 and the expression of immune checkpoint genes across multiple cancer types with poor prognoses. From a clinical standpoint, these findings offer a foundation for identifying and optimizing potential combination strategies to enhance the therapeutic effectiveness of immune checkpoint inhibitors and improve the prognosis of patients with LUAD.

## Data availability statement

The datasets presented in this study can be found in online repositories. The names of the repository/repositories and accession number(s) can be found in the article/**Supplementary Material**.

## Ethics statement

The studies involving humans were approved by the Institutional Ethics Committee of the Second Affiliated Hospital of Xuzhou Medical University. The studies were conducted in accordance with the local legislation and institutional requirements. The human samples used in this study were acquired from primarily isolated as part of your previous study for which ethical approval was obtained. Written informed consent for participation was not required from the participants or the participants’ legal guardians/next of kin in accordance with the national legislation and institutional requirements.

## Author contributions

ZH: Conceptualization, Data curation, Formal Analysis, Investigation, Methodology, Visualization, Writing – original draft, Writing – review & editing. XC: Methodology, Writing – review & editing. YW: Methodology, Writing – review & editing. JY: Methodology, Writing – review & editing. JL: Methodology, Writing – review & editing. WH: Methodology, Writing – review & editing. HM: Funding acquisition, Project administration, Supervision, Validation, Writing – review & editing.
