# Supplementary material for: SLC7A11 inhibits ferroptosis and downregulates PD-L1 levels in lung adenocarcinoma
Source: Front Immunol. 2024 Apr 9;15:1372215. doi: 10.3389/fimmu.2024.1372215 (PMC11035808; doi:10.3389/fimmu.2024.1372215)
Supplement: Supplementary file 1 [file DataSheet_1.docx]

Supplementary Material

# Supplementary Tables

Supplementary Table 1. Clinical characteristics of two clusters of LUAD patients.

|  | Feature | Cluster 1 | Cluster 2 | *p* |
| --- | --- | --- | --- | --- |
| Status | Alive | 244 | 85 |  |
|  | Dead | 114 | 73 | 0.002 |
| Age | Mean (SD) | 65.6 (10) | 64.7 (10.1) |  |
|  | Median (MIN, MAX) | 67 (33,87) | 65.5 (40,88) | 0.358 |
| Gender | FEMALE | 203 | 75 |  |
|  | MALE | 155 | 83 | 0.065 |
| Race | WHITE | 275 | 114 |  |
|  | Others | 83 | 44 | 0.268 |
| Smoking | Non-smoking | 68 | 21 |  |
|  | Smoking | 290 | 137 | 0.303 |
| pTNM_stage | I | 215 | 68 |  |
|  | II | 84 | 38 |  |
|  | III | 47 | 37 |  |
|  | IV | 12 | 15 | 0.002 |

Supplementary Table 2. Clinical characteristics of LUAD patients at the Second Affiliated Hospital of Xuzhou Medical University.

| No. | Age | Gender | Smoking | Pathological diagnosis | pTNM_stage |
| --- | --- | --- | --- | --- | --- |
| 1 | 38 | Female | No | LUAD | IV |
| 2 | 75 | Male | No | LUAD | III |
| 3 | 49 | Male | Yes | LUAD | IV |
| 4 | 64 | Female | No | LUAD | III |
| 5 | 66 | Female | No | LUAD | II |
| 6 | 77 | Female | No | LUAD | IV |
| 7 | 75 | Female | No | LUAD | I |
| 8 | 47 | Female | No | LUAD | IV |
| 9 | 76 | Male | Yes | LUAD | II |
| 10 | 74 | Male | Yes | LUAD | I |
| 11 | 65 | Female | Yes | LUAD | IV |
| 12 | 64 | Female | No | LUAD | III |
| 13 | 41 | Female | Yes | LUAD | III |
| 14 | 70 | Female | No | LUAD | III |
| 15 | 79 | Male | Yes | LUAD | IV |
| 16 | 79 | Male | No | LUAD | IV |
| 17 | 67 | Female | No | LUAD | II |
| 18 | 61 | Male | No | LUAD | II |
| 19 | 47 | Female | Yes | LUAD | IV |
| 20 | 41 | Male | Yes | LUAD | IV |
| 21 | 49 | Female | No | LUAD | III |
| 22 | 69 | Female | No | LUAD | I |
| 23 | 51 | Female | No | LUAD | I |
| 24 | 71 | Male | Yes | LUAD | III |
| 25 | 70 | Male | Yes | LUAD | II |
| 26 | 79 | Female | No | LUAD | IV |
| 27 | 65 | Female | No | LUAD | III |
| 28 | 58 | Female | No | LUAD | I |
| 29 | 59 | Male | Yes | LUAD | II |
| 30 | 75 | Male | Yes | LUAD | III |
| 31 | 77 | Male | No | LUAD | IV |
| 32 | 40 | Male | Yes | LUAD | II |
| 33 | 57 | Male | No | LUAD | II |
| 34 | 52 | Male | No | LUAD | I |
| 35 | 54 | Male | Yes | LUAD | III |
| 36 | 60 | Female | No | LUAD | II |
| 37 | 78 | Female | No | LUAD | II |

Supplementary Table 3. Sequence of the primers.

| Name |  | Sequence (5’->3’) |
| --- | --- | --- |
| GAPDH | Forward primer | TGTGGGCATCAATGGATTTGG |
|  | Reverse primer | ACACCATGTATTCCGGGTCAAT |
| SLC7A11 | Forward primer | GCGTGGGCATGTCTCTGAC |
|  | Reverse primer | GCTGGTAATGGACCAAAGACTTC |
| DPP4 | Forward primer | GGGTCACATGGTCACCAGTG |
|  | Reverse primer | TCTGTGTCGTTAAATTGGGCATA |
| GLS2 | Forward primer | GCCTGGGTGATTTGCTCTTTT |
|  | Reverse primer | CCTTTAGTGCAGTGGTGAACTT |
| PD-L1 | Forward primer | GCTGCACTAATTGTCTATTGGGA |
|  | Reverse primer | AATTCGCTTGTAGTCGGCACC |

Supplementary Table 4. The abbreviations of ferroptosis regulators.

| Abbreviation | Full name |
| --- | --- |
| ACSL4 | acyl-CoA synthetase long-chain family member 4 |
| ALOX15 | arachidonate 15-lipoxygenase |
| ATL1 | atlastin 1 |
| ATP5G3 | ATP synthase, H+ transporting, mitochondrial Fo complex subunit C3 |
| CARS | cysteinyl tRNA synthetase |
| CDKN1A | cyclin-dependent kinase inhibitor 1 |
| CISD1 | CDGSH iron sulfur domain 1 |
| CS | citrate synthase |
| DPP4 | dipeptidyl-dippeptidase-4 |
| EMC2 | ER membrane protein complex subunit 2 |
| FANCD2 | Fanconi anemia complementation group D2 |
| FDFT1 | farnesyl-diphosphate farnesyltransferase 1 |
| GLS2 | glutaminase 2 |
| GPX4 | glutathione peroxidase 4 |
| HSPA5 | heat shock protein family A member 5 |
| HSPB1 | heat shock protein beta 1 |
| MT1G | metallothionein-1G |
| NCOA4 | nuclear receptor coactivator 4 |
| NFE2L2 | nuclear factor, erythroid 2 like 2 |
| PCAT3 | lysophosphatidylcholine acyltransferase 3 |
| RPL8 | ribosomal protein L8 |
| SAT1 | spermidine/spermine N1-acetyltransferase 1 |
| SLC1A5 | solute carrier family 1 Member 5 |
| SLC7A11 | solute carrier family 7 member 11 |
| TFRC | transferrin receptor |

Supplementary Table 5. The abbreviations of multiple cancer types and immune-related checkpoints.

| Abbreviation | Full name |
| --- | --- |
| ACC | Adrenocortical carcinoma |
| BLCA | Bladder Urothelial Carcinoma |
| BRCA | Breast invasive carcinoma |
| CESC | Cervical squamous cell carcinoma and endocervical adenocarcinoma |
| CHOL | Cholangiocarcinoma |
| COAD | Colon adenocarcinoma |
| DLBC | Lymphoid Neoplasm Diffuse Large B-cell Lymphoma |
| ESCA | Esophageal carcinoma |
| GBM | Glioblastoma multiforme |
| HNSC | Head and Neck squamous cell carcinoma |
| KICH | Kidney Chromophobe |
| KIRC | Kidney renal clear cell carcinoma |
| KIRP | Kidney renal papillary cell carcinoma |
| LAML | Acute Myeloid Leukemia |
| LGG | Brain Lower Grade Glioma |
| LIHC | Liver hepatocellular carcinoma |
| LUAD | Lung adenocarcinoma |
| LUSC | Lung squamous cell carcinoma |
| MESO | Mesothelioma |
| OV | Ovarian serous cystadenocarcinoma |
| PAAD | Pancreatic adenocarcinoma |
| PCPG | Pheochromocytoma and Paraganglioma |
| PRAD | Prostate adenocarcinoma |
| READ | Rectum adenocarcinoma |
| SARC | Sarcoma |
| SKCM | Skin Cutaneous Melanoma |
| STAD | Stomach adenocarcinoma |
| TGCT | Testicular Germ Cell Tumors |
| THCA | Thyroid carcinoma |
| THYM | Thymoma |
| UCEC | Uterine Corpus Endometrial Carcinoma |
| UCS | Uterine Carcinosarcoma |
| UVM | Uveal Melanoma |
| PD-L1 | Programmed cell death 1 ligand 1 |
| CTLA4 | cytotoxic T-lymphocyte antigen 4 |
| LAG3 | lymphocyte-activation gene 3 |
| PDCD1LG2 | Recombinant Programmed Cell Death Protein 1 Ligand 2 |
| SIGLEC15 | sialic acid binding Ig-like lectin 15 |
| IDO1 | indoleamine 2,3-dioxygenase 1 |
| PDCD1 | programmed cell death 1 |
| TIM-3 | T cell immunoglobulin domain and mucin domain-3 |

# Supplementary Figures


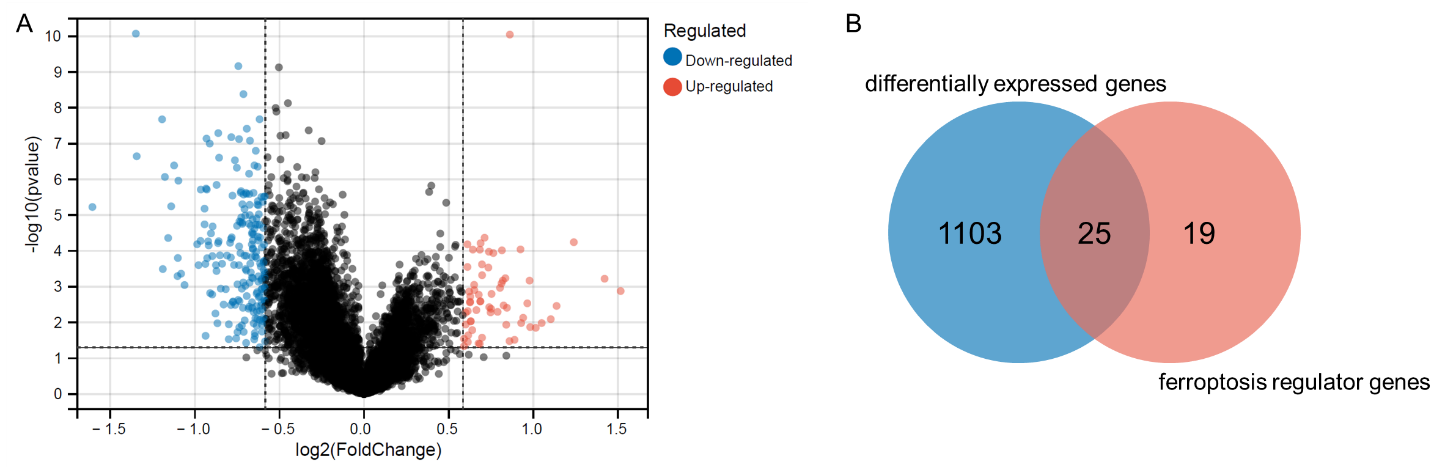


# Supplementary Figure 1. Screening for key ferroptosis regulators genes in LUAD. A. Volcano plot of differentially expressed genes in LUAD. B. Intersection of differentially expressed genes in LUAD and ferroptosis regulators genes.

#
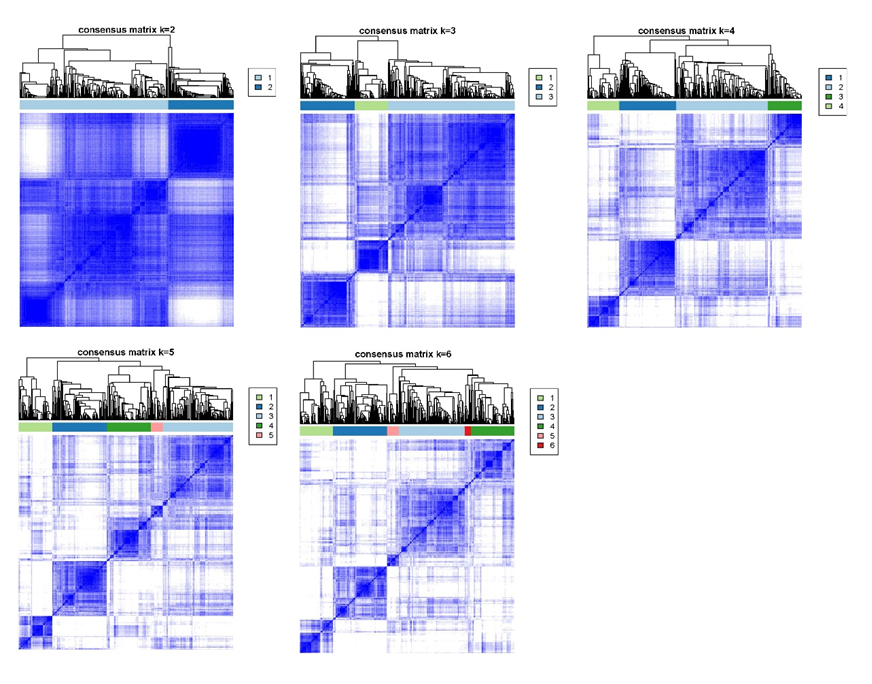


# Supplementary Figure 2. Consensus clustering for ferroptosis regulators in LUAD. Five heat maps exhibit the clustering matrix for ferroptosis regulators in LUAD patients for k =2, 3, 4, 5, and 6.


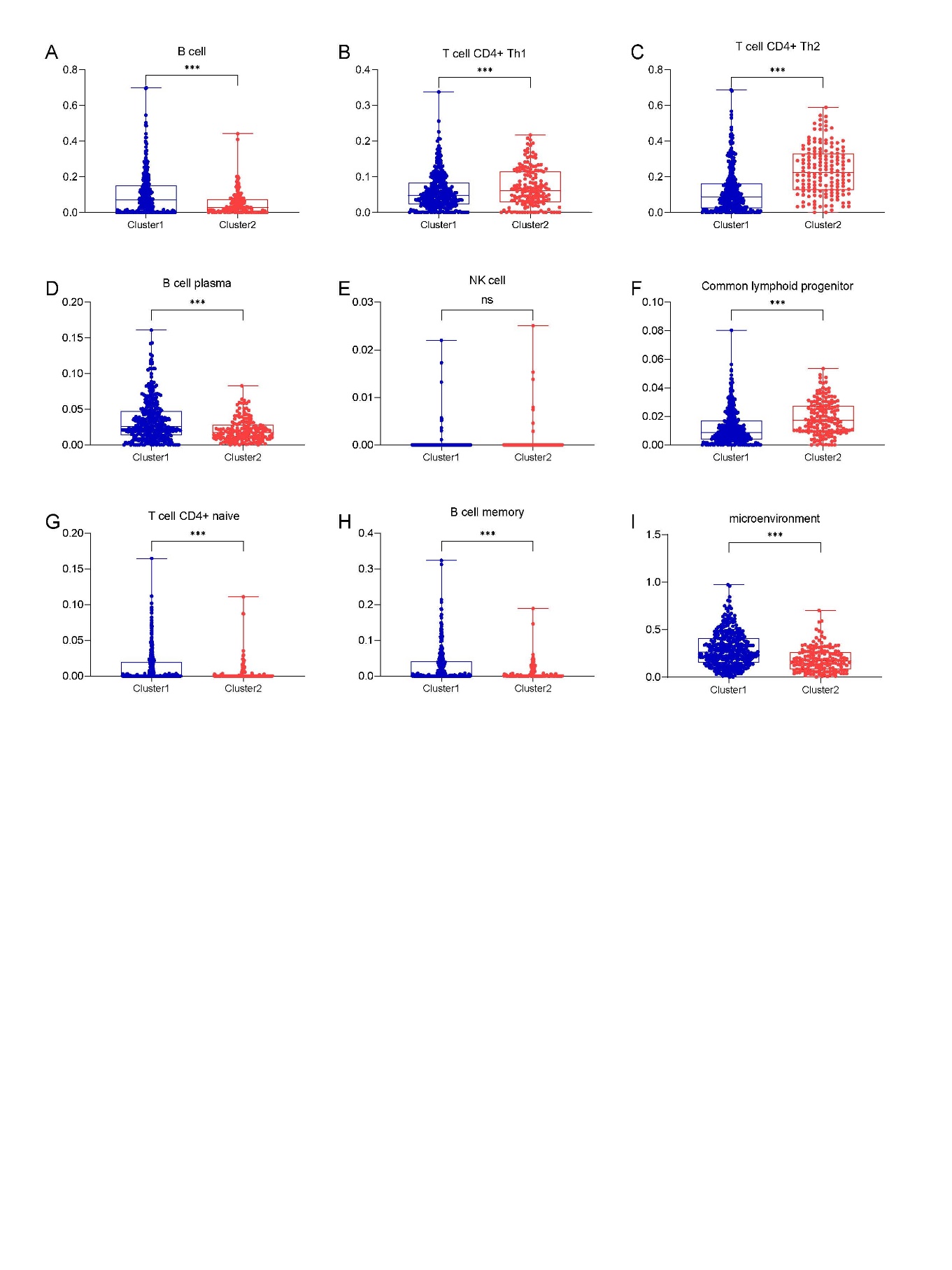


**Supplementary Figure 3.** The differences of immune cell infiltration levels in two LUAD subtypes.


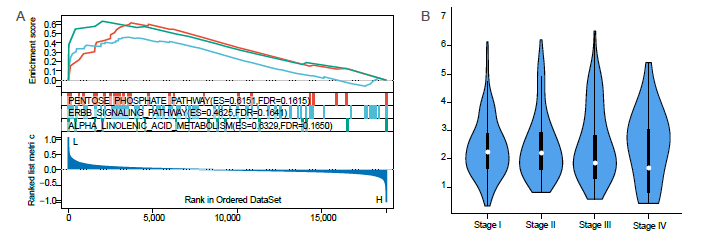
**Supplementary Figure 4.** A. Gene set enrichment analysis indicated that pentose phosphate, ERBB signaling and alpha linolenic acid metabolism pathways are significantly enriched in cluster 1. B. PD-L1 expression showed a decreasing trend with increasing LUAD disease stage, analysed in AGEPIA2.0.


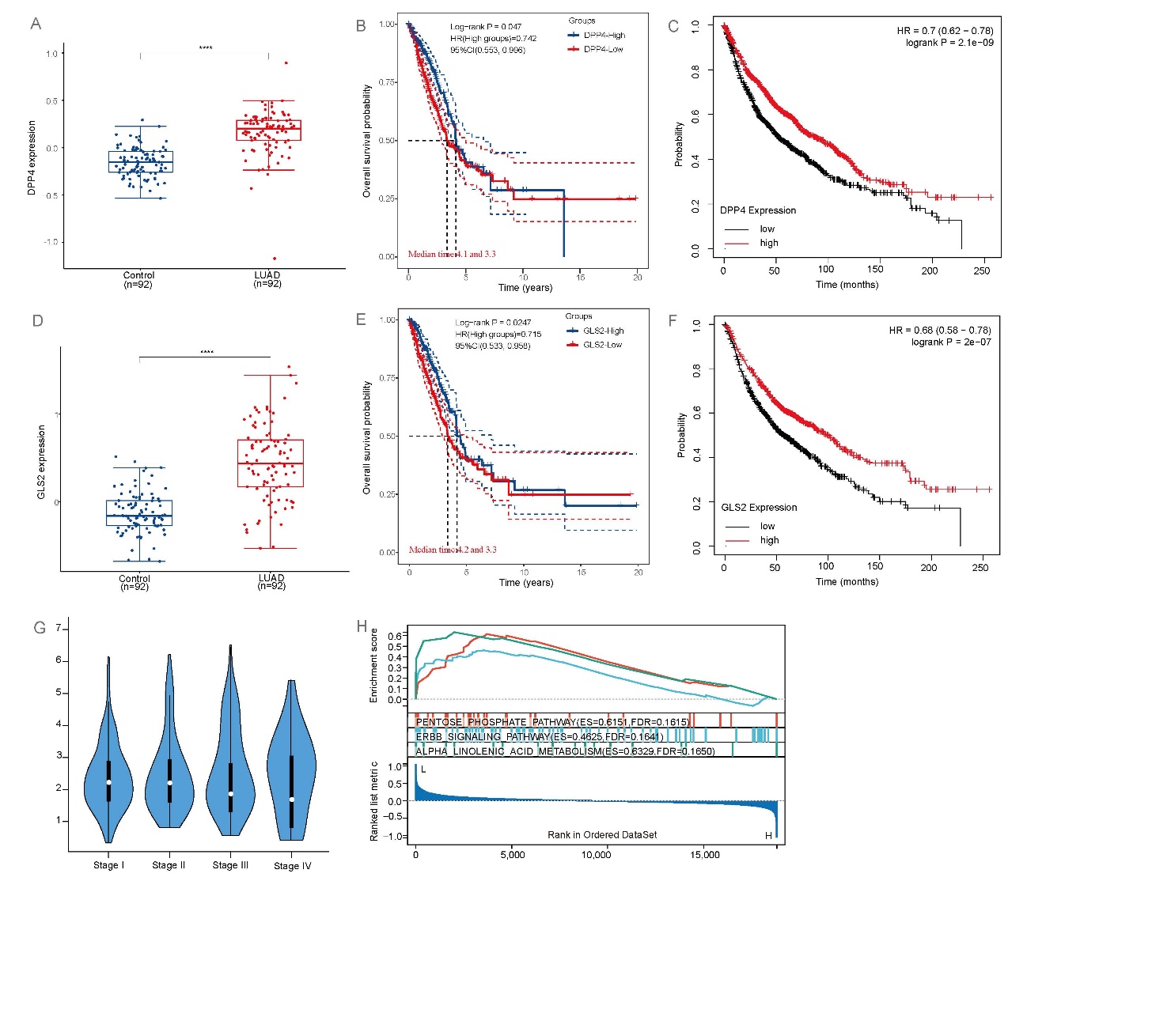


**Supplementary Figure 5.** Analysis of DPP4 and GLS2 gene expression in LUAD. A. DPP4 is highly expressed in LUAD patients in GSE46539 datasets. B. The Kaplan–Meier analysis of LUAD patients with high and low DPP4 expression level in the TCGA database. C. The Kaplan–Meier analysis of LUAD patients with high and low DPP4 expression level in Kaplan–Meier plotter database. D. GLS2 is highly expressed in LUAD patients in GSE46539 datasets. E. The Kaplan–Meier analysis of LUAD patients with high and low GLS2 expression level in the TCGA database. F. The Kaplan–Meier analysis of LUAD patients with high and low GLS2 expression level in Kaplan–Meier plotter database.


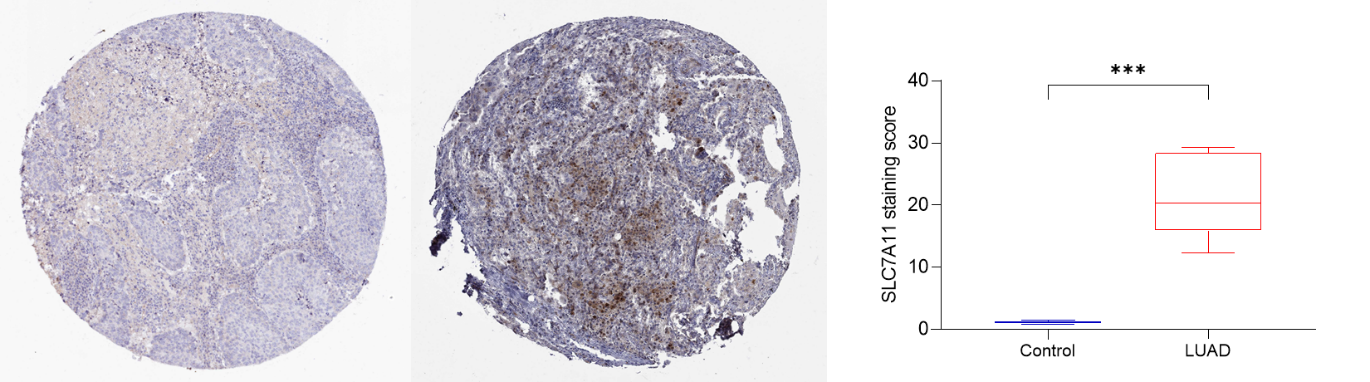


**Supplementary Figure 6.** Immunohistochemistry staining indicated that, compared with normal lung tissue (left), SLC7A11 was significantly elevated in LUAD tissue (right) in the human protein atlas (<https://www.proteinatlas.org/>, antibody HP HPA077839, ×10).


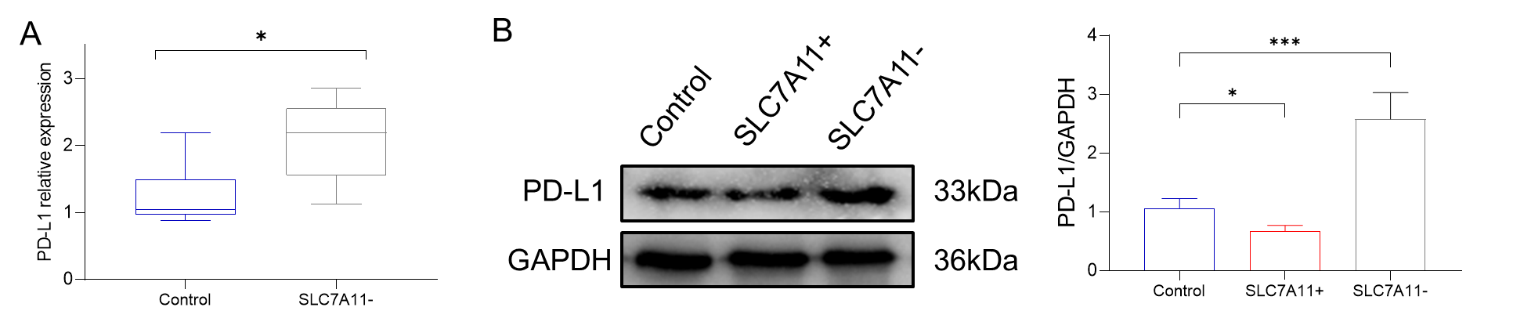


**Supplementary Figure 7.** A. Knockdown of SLC7A11 increased PD-L1 expression in A549 cells. B. After overexpression or knockdown of SLC7A11, the expression of PD-L1 in A549 cells was measured by western blot analysis.
